# Hippocampal Shape Is Associated with Memory Deficits in Temporal Lobe Epilepsy

**DOI:** 10.1002/ana.25762

**Published:** 2020-05-28

**Authors:** Tjardo S. Postma, Claire Cury, Sallie Baxendale, Pamela J. Thompson, Irene Cano‐López, Jane de Tisi, Jane L. Burdett, Meneka K. Sidhu, Lorenzo Caciagli, Gavin P. Winston, Sjoerd B. Vos, Maria Thom, John S. Duncan, Matthias J. Koepp, Marian Galovic

**Affiliations:** ^1^ Department of Clinical and Experimental Epilepsy University College London Queen Square Institute of Neurology London United Kingdom; ^2^ MRI Unit Epilepsy Society Chalfont St Peter United Kingdom; ^3^ GGZ inGeest Specialized Mental Health Care Amsterdam the Netherlands; ^4^ Department of Medical Physics and Biomedical Engineering University College London London United Kingdom; ^5^ University of Rennes, Inria, Inserm, CNRS, IRISA UMR 6074, Empenn team ERL U 1228, F‐35000 Rennes France; ^6^ Centre for Medical Image Computing University College London London United Kingdom; ^7^ Valencian International University Valencia Spain; ^8^ Department of Medicine, Division of Neurology Queen's University Kingston Canada; ^9^ Department of Neurology University Hospital Zurich Zurich Switzerland

## Abstract

**Objective:**

Cognitive problems, especially disturbances in episodic memory, and hippocampal sclerosis are common in temporal lobe epilepsy (TLE), but little is known about the relationship of hippocampal morphology with memory. We aimed to relate hippocampal surface‐shape patterns to verbal and visual learning.

**Methods:**

We analyzed hippocampal surface shapes on high‐resolution magnetic resonance images and the Adult Memory and Information Processing Battery in 145 unilateral refractory TLE patients undergoing epilepsy surgery, a validation set of 55 unilateral refractory TLE patients, and 39 age‐ and sex‐matched healthy volunteers.

**Results:**

Both left TLE (LTLE) and right TLE (RTLE) patients had lower verbal (LTLE 44 ± 11; RTLE 45 ± 10) and visual learning (LTLE 34 ± 8, RTLE 30 ± 8) scores than healthy controls (verbal 58 ± 8, visual 39 ± 6; *p* < 0.001). Verbal learning was more impaired the greater the atrophy of the left superolateral hippocampal head. In contrast, visual memory was worse with greater bilateral inferomedial hippocampal atrophy. Postsurgical verbal memory decline was more common in LTLE than in RTLE (reliable change index in LTLE 27% vs RTLE 7%, *p* = 0.006), whereas there were no differences in postsurgical visual memory decline between those groups. Preoperative atrophy of the left hippocampal tail predicted postsurgical verbal memory decline.

**Interpretation:**

Memory deficits in TLE are associated with specific morphological alterations of the hippocampus, which could help stratify TLE patients into those at high versus low risk of presurgical or postsurgical memory deficits. This knowledge could improve planning and prognosis of selective epilepsy surgery and neuropsychological counseling in TLE. ANN NEUROL 2020 **ANN NEUROL 2020;88:170–182**

## Introduction

Temporal lobe epilepsy (TLE) is the most frequent form of chronic focal epilepsy in adults, with up to 70% of TLE patients suffering from declarative memory problems.^1^ Many factors contribute to cognitive decline in TLE, including the neuronal, physical, and psychological impact of seizures, comorbidities, head trauma, and medical treatment. Despite its impact on patients' quality of life, several key questions regarding cognitive comorbidities in TLE remain unanswered.

First, how strongly are memory functions lateralized in TLE? The traditional material‐specific model states that verbal deficits are usually observed in epilepsy affecting the dominant (usually left) hemisphere,^2^ whereas visual memory is typically affected by epilepsy in the nondominant (usually right) hemisphere.^3^ This concept is one of the cornerstones of presurgical planning and provides a framework for assessing cognitive risks associated with epilepsy surgery and postoperative outcome. However, verbal memory deficits are not restricted to left TLE (LTLE), as right TLE (RTLE) patients also display poor performance on verbal memory tests, suggesting a more bilateral framework.^4^ Following anterior temporal lobe resections (ATLRs), memory functions decline in one‐quarter of surgically treated TLE patients.^5^ Verbal memory decline can also be observed after right temporal lobe removal, although less frequently than after left temporal lobe resections.^5^ Several functional magnetic resonance imaging (fMRI) studies suggest a more bilateral dynamic functionality between both temporal lobes.^6^, ^7^, ^8^

Second, why do some patients with TLE have memory impairments whereas others do not? Most cognitive difficulties are already detectable at or even before the onset of seizures, suggesting an important role of the underlying structural and cellular pathology.^1^ Hippocampal volume reductions have been linked to verbal and visual memory deficits,^9^ but it remains uncertain whether certain areas of the hippocampus specifically contribute to verbal and visual memory formation, and consequently, whether particular subtypes of hippocampal sclerosis (HS) are associated with presurgical memory deficits or carry a higher risk of postsurgical memory decline.^10^

Lastly, is there a subregionally specific contribution of the hippocampus to verbal and visual memory formation? Various quantitative histology studies have assessed neuronal cell counts on hippocampal specimens obtained during temporal lobe resections but produced conflicting findings (Table S1).^10^, ^11^, ^12^, ^13^, ^14^, ^15^, ^16^, ^17^, ^18^, ^19^, ^20^

Histological neuronal cell counts have 2 major limitations. Histopathology only evaluates tissue from a limited number of slices of the hippocampus, restricting the method's ability to make spatial inferences on the location of the findings. Additionally, the contralateral hippocampus cannot be investigated.

To overcome some of these limitations, we analyzed alterations of hippocampal morphology using noninvasive surface shape analysis on high‐resolution MRI,^21^ allowing analysis of both resected and nonresected tissue. Specifically, we aimed to (1) determine the localization and extent of surface displacements within the left and right hippocampi that are associated with verbal and visual memory performance in TLE and (2) define whether the identified imaging patterns can be used to stratify TLE patients into those at high or low risk of presurgical and postsurgical memory deficits. Such knowledge would be crucial to guide neuropsychological counseling before temporal lobe removal and might lead to recommendations for tailored resections.

## Subjects and Methods

### Participants

We identified consecutive TLE patients undergoing epilepsy surgery at the National Hospital for Neurology and Neurosurgery (NHNN; London, UK) and 39 age‐ and sex‐matched healthy volunteers. The details of our ongoing epilepsy cohort study have been described previously.^22^ We included a subset of 145 patients with medically refractory TLE, as determined by multidisciplinary presurgical evaluation before undergoing temporal lobe resections. All participants had preoperative high‐resolution structural T1‐weighted MRI on the same scanner and received a standardized multidisciplinary preoperative evaluation including long‐term video‐electroencephalographic telemetry, and neuropsychiatric and neuropsychological evaluations. We excluded those with lesions other than HS that could alter hippocampal morphology (eg, cavernoma, dysplasia, or tumors affecting the medial temporal lobe) and scans of insufficient quality (ie, subject movement or technical artifacts). Participants with lesions that did not affect the mesial temporal lobe were not excluded.

In addition, we included a validation set of 55 independent unilateral refractory TLE patients (30 LTLE, age = 37.5 ± 12.0 years, 29 female) evaluated at NHNN and meeting the criteria described above.

The standard ATLR consisted of the removal of the temporal pole and opening of the temporal horn, followed by en bloc resection of the hippocampus with a posterior resection margin at the midbrainstem level. Typically, the anterior–posterior extent of the temporal lobe resection as measured from the temporal pole to the posterior margin of resection is 30% and 35% of the distance from the temporal pole to the occipital pole after left and right ATLR, respectively. Only the anterior part of the hippocampus is resected during this procedure, and parts of the body and the tail remain as a postsurgical remnant.^23^ The operation being performed by the same neurosurgeon resulted in little variation of the temporal neocortical extent of the resection. The study was classified as a service evaluation involving further anonymized analysis of previously acquired clinical data, not requiring individual consent. Healthy controls provided written consent as part of previous studies approved by the local research ethics committee.

### Neuropsychological Evaluation

Verbal and visual memory was assessed preoperatively and 1 year postoperatively using the Adult Memory and Information Processing Battery (AMIPB).^24^ Data on postoperative (1 year) follow‐up was available in 109 patients in the main dataset (55/76 with LTLE and 54/69 with RLTE, *p* = 0.41) and in 24 patients in the validation cohort (14/30 with LTLE and 10/25 with RTLE, *p* = 0.79). List and Design learning was used for memory assessment.^25^ During this task, subjects will read a list of 15 words 5 times.^26^ After each reading, the subject is asked to immediately recall as many words as possible, and the total number of correctly recalled items is used as an indicator of verbal learning. To assess visual memory, subjects are presented with a graphical design 5 times, with learning being tested after each presentation. Delayed recall has been tested as the number of correctly recalled items following a distraction task.

Change in neuropsychological performance 1 year after epilepsy surgery was measured as the difference in pre‐ and postsurgical memory *z* scores. Significant memory decline was defined as decline below the 90% reliable change index (RCI) as described previously.^5^ The RCI reflects meaningful memory decline adjusted for test reliability and practice effects in a test–retest setting.

### MRI Acquisition

MRI data for the development cohort were collected between September 2005 and August 2012 on a 3T Signa HDx Scanner (GE, Milwaukee, WI) at the Epilepsy Society. A coronal T1‐weighted 3‐dimensional (3D) fast spoiled gradient echo with repetition time (TR)/echo time (TE)/inversion time (TI) = 6.6/2.8/450 milliseconds, matrix = 256 × 256 × 178, field of view (FOV) = 24 × 240 × 196mm, voxel size = 0.9375 × 0.9375 × 1.1mm was acquired in all subjects.

In the validation cohort, subjects underwent imaging on a 3T GE Discovery MR750. A 3D T1‐weighted inversion‐recovery fast spoiled gradient recalled echo (TE/TR/TI = 3.1/7.4/400 milliseconds, FOV = 224 × 256 × 256mm, matrix 224 × 256 × 256, parallel imaging acceleration = 2) was acquired in all validation subjects.

### Hippocampal Segmentation

First, we used Hipposeg^27^ (http://niftyweb.cs.ucl.ac.uk/program.php?p=HIPPOSEG) to automatically extract the initial hippocampal masks.^28^ Hipposeg delineates the hippocampus with no more variability than seen between expert human raters and is robust to atrophic hippocampi. Second, one blinded rater (T.S.P.) received anonymized hippocampal masks and corrected misclassifications (eg, inclusion of the parahippocampal gyrus in the hippocampal segmentation) according to a well‐established protocol^29^ using ITK‐SNAP.^30^

To assess intrarater variability of this combined manual‐automated approach, one blinded rater manually corrected Hipposeg segmentation in randomly selected 10 TLE patients on two different occasions 3 months apart and compared the resulting masks using Dice coefficients.^31^ To determine inter‐rater variability, a second blinded rater corrected Hipposeg segmentations of 10 randomly selected TLE patients using the same segmentation protocol. A high intrarater (0.98 ± 0.01) and inter‐rater (0.96 ± 0.02) reliability demonstrated a high consistency of the combined manual–automated method, exceeding the reliability reported for an entirely manual method (intrarater 0.89 ± 0.02, inter‐rater 0.83 ± 0.02).^27^

Hippocampal volumes were corrected for total intracranial volume (TIV) as described previously.^27^ TIV was calculated using a parcellation algorithm based on geodesic information flows.^32^

### Hippocampal Shape Analysis

3D surface meshes were extracted from binary hippocampal segmentations and parameterized with a spherical harmonics point distribution model (SPHARM‐PDM).^21^, ^33^ In short, to ensure spherical topology of hippocampal segmentations, uneven boundaries were minimally smoothed while the original binary surface was used as a constraint ensuring marginal loss of ±3 voxels of the original surface. Subsequently, these surfaces were represented by spherical harmonics (SPHARM), which were then sampled onto triangulated surfaces (SPHARM‐PDM). We generated a mean shape from 39 healthy controls to which all hippocampal surfaces were aligned. Hippocampal shapes were visually checked for both surface mesh and alignment failures. Displacement values were generated using a point to mesh approach calculating the shortest perpendicular distance between the mean template surface and each point on an individual's hippocampal surface mesh. An inward displacement (negative displacement value) typically corresponds to atrophy, outward displacement (positive displacement value) to hypertrophy. All preprocessing was done separately for left and right hippocampi.

**FIGURE 1:**
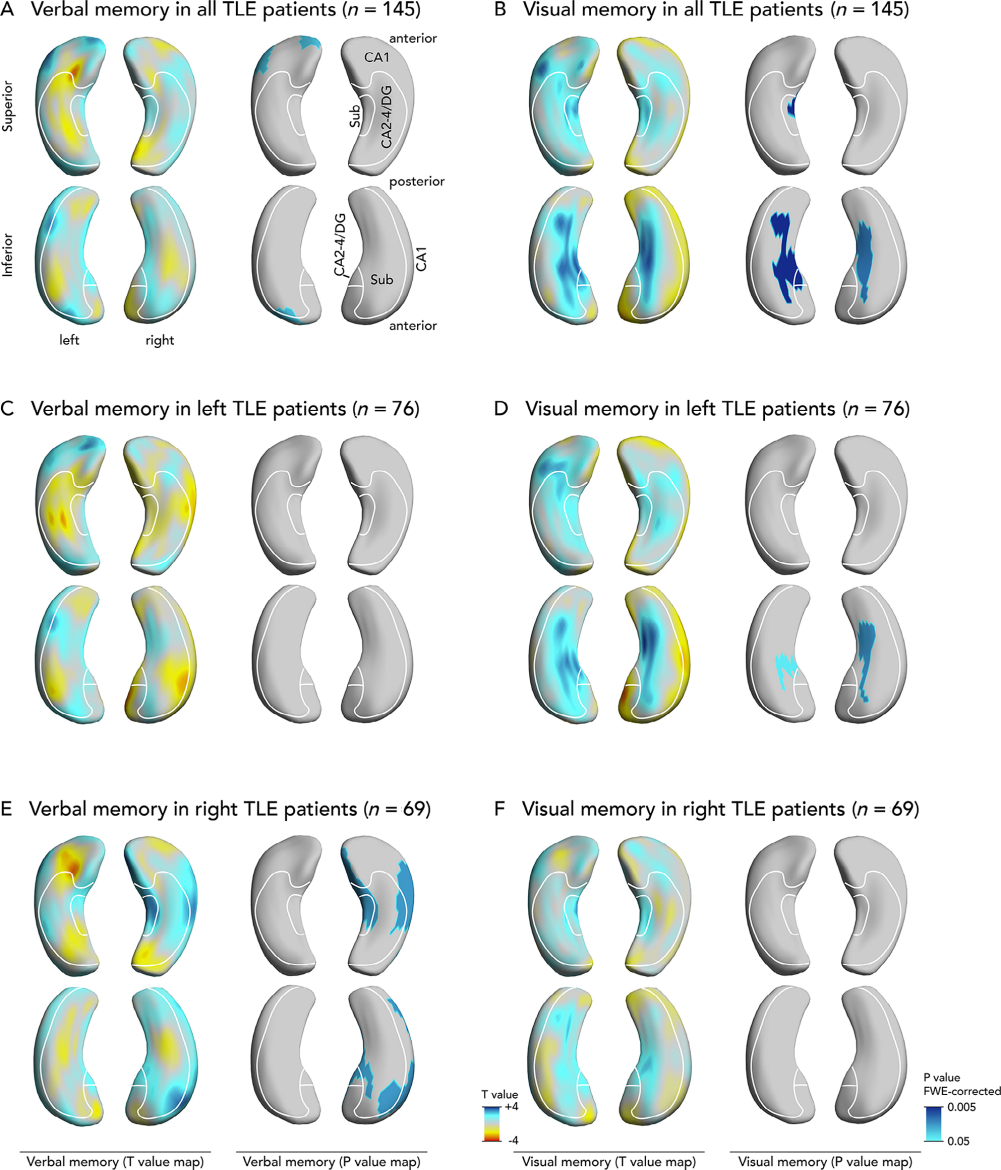
Association of hippocampal shape alterations with presurgical verbal and visual memory in temporal lobe epilepsy (TLE). Upper panels show findings common to both left and right TLE, correcting for epilepsy lateralization (A, verbal memory; B, visual memory). Also displayed are separate analyses of left (C, verbal memory; D, visual memory) and right (E, verbal memory; F, visual memory) TLE subgroups. Each panel shows T‐value maps on the left, representing deformations related to memory scores (blue colors: more atrophy related to worse memory). Significant *p* values are displayed on the right thresholded to *p* < 0.05 corrected for multiple comparisons using random field theory. The right and left hippocampi are visualized from a superior and an inferior perspective. An approximation of major hippocampal subregional boundaries is overlaid on hippocampal surfaces, description in A. CA = cornu ammonis; DG = dentate gyrus; FWE = familywise error; Sub = subiculum.

**FIGURE 2:**
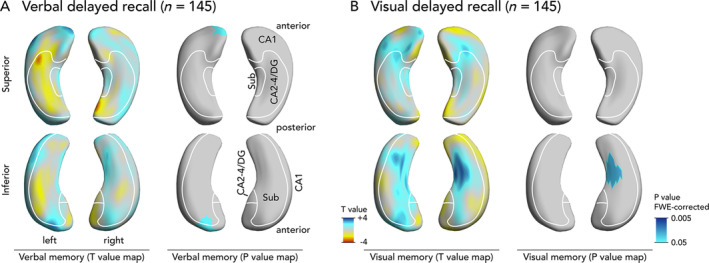
Association of hippocampal shape alterations with presurgical verbal and visual delayed recall in temporal lobe epilepsy (TLE). Panels show findings common to both left and right TLE, correcting for epilepsy lateralization (A, verbal delayed recall; B, visual delayed recall). Each panel shows T‐value maps on the left, representing deformations related to memory scores (blue colors: more atrophy related to worse memory). Significant *p* values are displayed on the right thresholded to *p* < 0.05 corrected for multiple comparisons using random field theory. The right and left hippocampi are visualized from a superior and an inferior perspective. An approximation of major hippocampal subregional boundaries is overlaid on hippocampal surfaces, description in A. CA = cornu ammonis; DG = dentate gyrus; FWE = familywise error; Sub = subiculum.

To enhance the interpretation of hippocampal surface maps, several groups have defined heuristic boundaries between hippocampal subregions.^34^, ^35^, ^36^ This approach provides a conceptually sound approximation, because all subfields reach the surface, except CA4, which is hidden in depth.^36^ With this in mind, we defined major hippocampal subregional boundaries using surface projections of histological data (Figs 1 and 2).

### Statistical Analysis

We statistically compared pointwise displacement values on hippocampal surfaces using fixed‐effect linear models implemented in SurfStat (http://www.math.mcgill.ca/keith/surfstat/). Two independent variables (verbal memory and visual memory) were analyzed in 3 groups (all TLE patients and the subgroups of LTLE and RTLE).

We first compared all TLE patients in a single analysis while controlling for epilepsy lateralization. This allowed us to detect findings that are common to both LTLE and RTLE, irrespective of lateralization. We adjusted the analyses for TIV and lateralization of epilepsy to correct for different patterns of network disruption occurring in LTLE and RTLE.

To analyze the association of the presurgical shape of the to‐be‐resected hippocampus with postsurgical memory outcome, we restricted the postsurgical memory analyses only to the ipsilateral hippocampus (ie, left for left‐sided resections, right for right‐sided resections). The outcome parameter used was the change in *z* scores between pre‐ and 1‐year postsurgical memory assessments, and the analyses were corrected for age at onset of habitual seizures and residual seizures after surgery.

We report findings corrected for multiple comparisons using random field theory for nonisotropic images on a cluster level,^37^ thresholded to a corrected *p* < 0.05.

The effect size is shown on T‐value maps. A higher T value (blue color) signifies a positive correlation, that is, the association between a regional inward deformation (atrophy) and lower memory performance. A negative T value represents a negative correlation, that is, the association of outward deformations (hypertrophy) with lower memory performance.

We also aimed to determine the prognostic value of the significant shape patterns. On an individual level, we determined the predictive value of deformations in these clusters using the area under the receiver operating characteristics curve (AUC). On a group level, we divided patients into those with or without significant inward deviations (ie, below the 5th percentile of values estimated in 39 healthy controls). We compared poor presurgical performance and the risk of poor postsurgical memory performance between these imaging‐based patient subgroups using logistic regression and provide bootstrapped confidence intervals (CIs) to improve generalizability. In addition, we used logistic regression and calculated the AUC to determine the association of hippocampal shape patterns with memory performance in the validation cohort.

Demographic data and volumetric findings are reported as n (%) or mean ± standard deviation. We compared hippocampal volumes between groups with the *t* test for independent samples. We correlated hippocampal volumes with neuropsychological scores using the Pearson correlation coefficient^38^ corrected for TIV, age at seizure onset, and seizure frequency. Calculations were done in SPSS (v24; IBM, Armonk, NY).

## Results

### Participant Demographics

We included 76 patients with LTLE (45 female, age = 38 ± 12 years), 69 patients with RTLE (40 female, age = 39 ± 11 years), and 39 controls (25 female, age = 36 ± 11 years). There were no between‐group differences in age (*t* = −1,337, *p* = 0.183) or sex (*t* = 0.617, *p* = 0.538). Detailed participant characteristics are displayed in Table 1.

**TABLE 1:** Participant Characteristics

Characteristic	All Patients, n = 145	LTLE, n = 76	RTLE, n = 69	Healthy Controls, n = 39
Sex				
F	85 (59%)	45 (59%)	40 (58%)	25 (64%)
M	60 (41%)	31 (41%)	29 (42%)	14 (36%)
Presurgical seizures				
SPS	76 (52%)	41 (54%)	35 (51%)	N/A
CPS	139 (96%)	73 (96%)	66 (96%)	N/A
SGS	102 (70%)	53 (70%)	49 (71%)	N/A
CPS frequency, per mo	16 ± 83	11 ± 14	23 ± 120	N/A
SGS frequency, per mo	0.8 ± 2.0	1.0 ± 2.6	0.5 ±1.0	N/A
Age and duration, yr				
Age at scan	38 ± 12	38 ± 12	39 ± 11	36 ± 11
Age at seizure onset	16 ± 12	16 ± 12	15 ± 11	N/A
Epilepsy duration at scan	23 ± 13	22 ± 3	23 ± 14	N/A
Pathology				
HS	100 (69%)	52 (68%)	48 (70%)	N/A
Cavernoma	8 (6%)	4 (5%)	4 (6%)	N/A
DNT	9 (6%)	5 (7%)	4 (6%)	N/A
Focal cortical dysplasia	2 (1%)	2 (3%)	0 (0%)	N/A
Gliosis	7 (5%)	5 (7%)	2 (3%)	N/A
Other^a^	7 (5%)	2 (3%)	5 (7%)	N/A
No abnormality	12 (8%)	6 (8%)	6 (9%)	N/A
Neuropsychometry before surgery				
Verbal memory	44.2 ± 10.7^b^	43.5 ± 11.3^b^	45.2 ± 9.9^b^	58.3 ± 8.3
Visual memory	31.8 ± 8.0^b^	33.5 ± 7.9^b^	29.9 ± 7.8^b^ ^,^ ^c^	38.8 ± 5.6
Verbal memory recall	8.5 ± 3.4^b^	7.6 ± 3.7^b^ ^,^ ^c^	9.5 ± 2.8^b^	12.4 ± 2.5
Visual memory recall	6.3 ± 2.6^b^	6.6 ± 2.5^b^	6.0 ± 2.7^b^	8.1 ± 1.5
Neuropsychometry 1 yr after surgery^c^				
Change in verbal memory	−3.1 ± 11.3	−6.8 ± 11.7^c^	0.6 ± 9.6	N/A
Change in visual memory	−1.2 ± 7.0	−2.1 ± 7.7	−0.2 ± 6.1	N/A
RCI decline in verbal memory	19 (17%)	15 (27%)^c^	4 (7%)	N/A
RCI decline in visual memory	8 (7%)	5 (9%)	3 (6%)	N/A
Hippocampal volume, cm^3^				
Left hippocampus	2.6 ± 0.5	2.3 ± 0.5^b^ ^,^ ^c^	2.9 ± 0.3	2.9 ± 0.2
Right hippocampus	2.7 ± 0.6	3.03 ± 0.3^b^	2.4 ± 0.6^b^ ^,^ ^c^	2.9 ± 0.2

a Other: gliosis, focal cortical dysplasia, undiagnosed.

b Significant (*p* < 0.05) difference compared to controls.

c Neuropsychometry data 1 year after epilepsy surgery were not available in 36 patients.

CPS = complex partial seizures; DNT = dysembryoplastic neuroepithelial tumor; F = female; HS = hippocampal sclerosis; LTLE = left temporal lobe epilepsy; M = male; N/A = not applicable; RCI = reliable change index indicating memory decline below the 5th percentile of the expected change on a follow‐up examination; RTLE = right temporal lobe epilepsy; SGS = secondary generalized seizures; SPS = simple partial seizures.

Both RTLE and LTLE patients had lower verbal (LTLE *t* = 8.495, RTLE *t* = 7.867) and visual (LTLE *t* = 4.158, RTLE *t* = 6.821) memory scores than healthy controls (*p* < 0.001; see Table 1). Both groups also had lower verbal (LTLE *t* = 7.3, RTLE *t* = 5.4) and visual (LTLE *t* = 3.5, RTLE *t* = 4.5) delayed recall scores than healthy controls (*p* ≤ 0.001; see Table 1). There were no differences in mean verbal memory between patient groups (RTLE 45.2 vs LTLE 43.5, *t* = 0.983, *p* = 0.33), but RTLE patients had lower visual memory scores than LTLE patients (RTLE 29.9 vs LTLE 33.5, *t* = −2.722, *p* = 0.007).

Postsurgical (1 year) verbal memory decline was more common after left compared to right temporal lobe removal (mean change in verbal memory scores: LTLE −6.8 vs RTLE 0.6, *p* = 0.001; decline on the RCI: LTLE 27% vs RTLE 7%, *t* = 3.550, *p* = 0.006). There were no significant changes in visual memory after left or right ATLR.

There was no difference in memory scores between lesional and nonlesional patients (Table S2). Data on language lateralization and its association with pre‐ and postsurgical memory performance in LTLE are given in Table S3.

### Volumetric Results

The ipsilateral hippocampus was significantly smaller in both LTLE (*t* = −7.438) and RTLE (*t* = −6.467) patients compared to controls (*p* < 0.001; see Table 1). The right hippocampus was larger in LTLE than in controls (*t* = 2.821, *p* = 0.006).

For all TLE patients, a greater left hippocampal volume weakly correlated with better verbal memory (*r* = 0.16, *p* = 0.047) and greater right hippocampal volume with better visual memory (*r* = 0.18, *p* = 0.03) before surgery (Table 2). In RTLE patients, increased left, that is, contralateral, hippocampal volume correlated with better visual memory before surgery (*r* = 0.24, *p* = 0.04).

**TABLE 2:** Correlation between Hippocampal Volume and Memory Scores before and after Epilepsy Surgery

	Before Surgery		1 Year after Surgery^a^	
Patient Group	Verbal Memory	Visual Memory	Change in Verbal Memory	Change in Visual Memory
All patients, N = 145				
Left hv	0.17^b^	0.05	0.25^c^	0.02
Right hv	0.00	0.19^b^	−0.18	0.00
LTLE, n = 76				
Left hv	0.15	0.18	0.17	−0.09
Right hv	−0.18	−0.02	0.18	0.18
RTLE, n = 69				
Left hv	0.16	0.27^b^	−0.13	−0.05
Right hv	0.12	0.15	−0.01	0.11

a Neuropsychometry data 1 year after epilepsy surgery were not available in 30 patients.

b *p* < 0.05.

c *p* < 0.01.

hv = hippocampal volume; LTLE = left temporal lobe epilepsy; RTLE = right temporal lobe epilepsy.

The presurgical volume of the left hippocampus predicted postsurgical change in verbal memory for all patients with TLE (*r* = 0.28, *p* = 0.003). Although significant, these correlation coefficients (*r* < 0.30) only suggest a weak linear relationship between overall hippocampal volume and neuropsychological performance.

### Hippocampal Shape and Presurgical Memory

#### All TLE Patients (n = 145)

We compared presurgical memory performance with hippocampal shape patterns that were common to both LTLE and RTLE while correcting for epilepsy lateralization.

Atrophy of the left superolateral hippocampal head was associated with decreased verbal memory (see Fig 1A). Atrophy of the inferomedial hippocampal surface bilaterally was associated with worse visual memory (see Fig 1B).

We also compared hippocampal shape patterns with delayed recall (see Fig 2). Delayed verbal recall was associated with atrophy of the left superolateral hippocampal head, and delayed visual recall was associated with atrophy of the right inferomedial hippocampal surface.

#### LTLE (n = 76)

In LTLE patients, we did not detect a significant pattern of hippocampal shape alterations associated with verbal memory (see Fig 1). However, the pattern seen on the T‐value map in LTLE patients was largely similar to that observed in all TLE patients, without any clusters reaching significance. We observed that bilateral atrophy of the inferomedial hippocampal surface was significantly associated with poorer visual memory performance.

#### RTLE (n = 69)

In RTLE, increased atrophy of the medial and lateral surface of the ipsilateral hippocampus corresponded to decreased mean verbal memory scores (see Fig 1E). No significant hippocampal shape patterns were linked to visual memory (see Fig 1F).

### Hippocampal Shape and Postsurgical Memory

**FIGURE 3:**
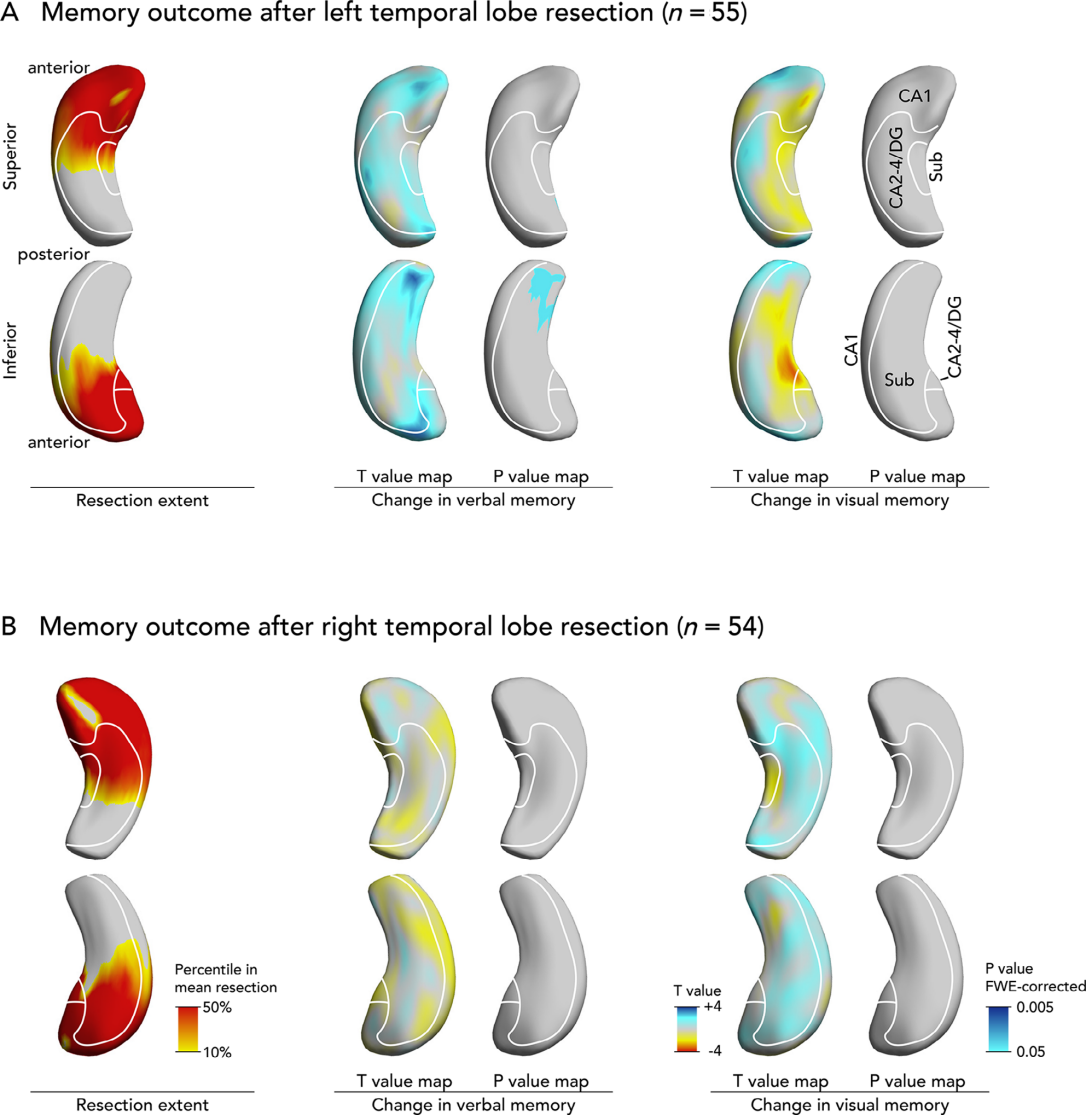
Association of presurgical hippocampal shape alterations with verbal and visual memory decline after anterior temporal lobe resection (ATLR). Displayed are results after left (A) and right (B) ATLRs. The mean extent of hippocampal resection projected on the hippocampal surface is shown on the left. T‐value maps represent deformations related to decline in memory scores (blue colors: more presurgical atrophy related to postsurgical worsening of memory). Significant *p* values are displayed on the right thresholded to *p* < 0.05 corrected for multiple comparisons using random field theory. The right and left hippocampi are visualized from a superior and an inferior perspective. An approximation of major hippocampal subregional boundaries is overlaid on hippocampal surfaces, description in A. CA = cornu ammonis; DG = dentate gyrus; FWE = familywise error; Sub = subiculum.

Preoperative atrophy of the left hippocampal tail was associated with decline in verbal memory 1 year after anterior left temporal lobe resection (Fig 3A). This part of the hippocampus will not be resected (see Fig 3A left).

There were no significant shape patterns predicting visual memory outcome after left temporal resection (see Fig 3A), or visual or verbal memory outcome after right temporal resections (see Fig 3B).

### Associations of Hippocampal Shape Patterns

**FIGURE 4:**
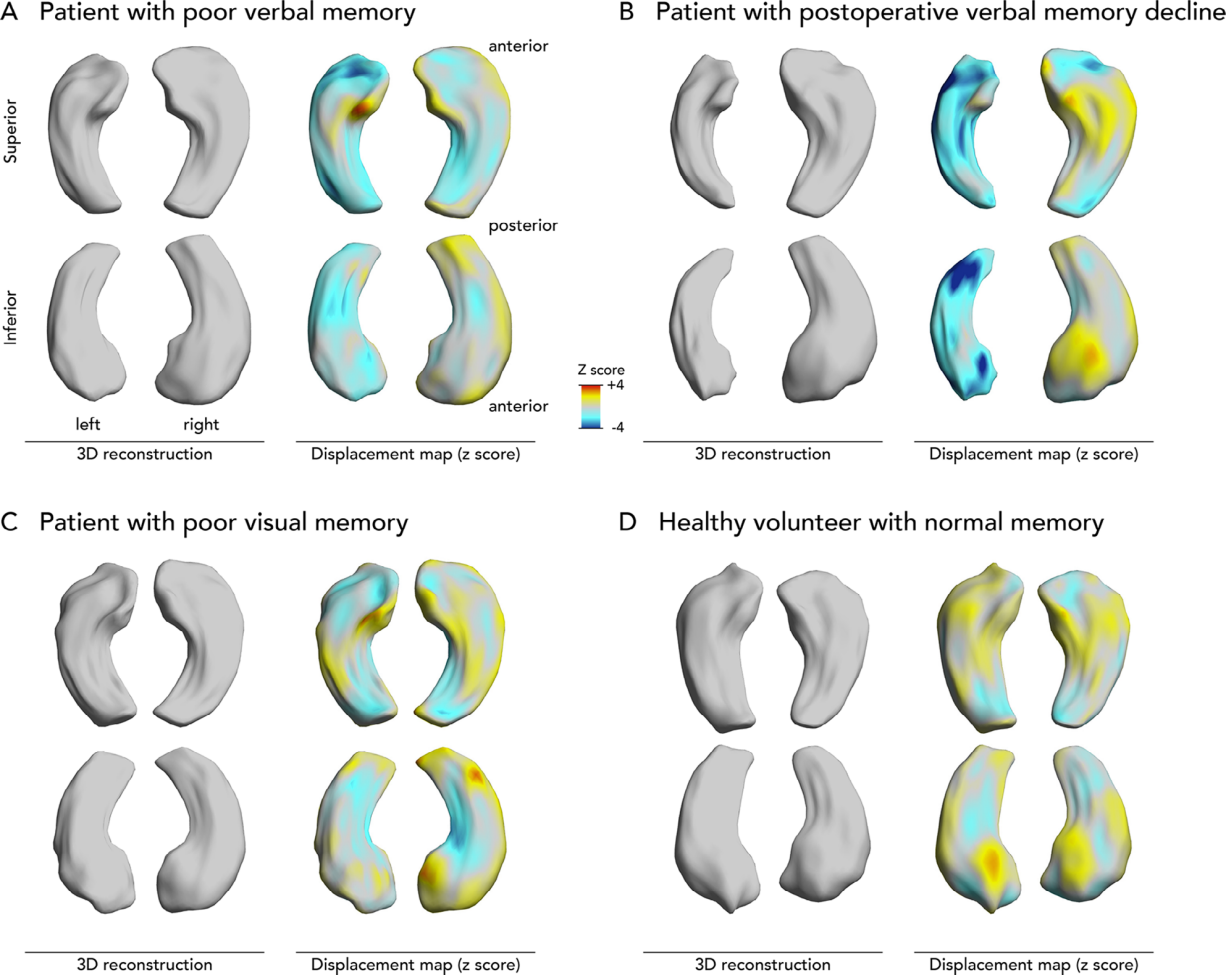
Three‐dimensional (3D) reconstructions of hippocampi in example cases. Displayed are 3D reconstructions of the hippocampal surface. The *z* scores of inward (blue, ie, atrophy) or outward (yellow/red, ie, hypertrophy) displacements from a mean normal hippocampal template are projected onto these surfaces. (A) Hippocampi of a 41‐year‐old female with left temporal lobe epilepsy with poor presurgical verbal memory (25 points) and slightly impaired visual memory (30 points) but no verbal memory decline after surgery (35 points). She had left hippocampal atrophy mainly affecting the left hippocampal head. (B) Hippocampi of a 63‐year‐old female with left temporal lobe epilepsy with slightly impaired presurgical verbal (41 points) and visual (26 points) memory and verbal memory decline following surgery (24 points). She had left hippocampal atrophy affecting the left hippocampal head and tail. (C) Hippocampi of a 65‐year‐old female with right temporal lobe epilepsy with normal presurgical verbal memory (44 points) and poor visual memory (12 points) but no verbal or visual memory decline after surgery (49 and 10 points, respectively). Despite normal hippocampal volumes, there was localized atrophy that affected the inferomedial hippocampal surface mainly on the right. (D) Hippocampi of a 30‐year‐old healthy male with good verbal and visual memory performance (73 and 43 points, respectively).

3D reconstructions of the hippocampi of 4 example cases are presented in Figure 4.

#### Verbal Memory before Surgery

Patients with (n = 55) compared to those without (n = 90) significant atrophy in the left superolateral hippocampal head (see Fig 1A) had a 2.9‐fold (95% CI = 1.2–6.8) higher risk of poor verbal memory (35% vs 16%, *p* = 0.008). Atrophy here was a significant predictor of poor verbal memory (AUC = 0.66, *p* = 0.004), and this association remained significant when controlling for presence of HS (*p* = 0.009). This pattern was, however, not predictive of poor visual memory performance (24% vs 13%, *p* = 0.18).

In the validation cohort, patients with (n = 13) compared to those without (n = 42) significant atrophy in the left superolateral hippocampal head had a 3.3‐fold (95% CI = 0.7–14.8; 31% vs 12%) higher risk of poor verbal memory with an AUC of 0.74 (*p* = 0.003).

#### Visual Memory before Surgery

Patients with (n = 33) as opposed to those without (n = 112) significant atrophy of the bilateral inferomedial hippocampal surface (see Fig 1B) had a 9.5‐fold (95% CI = 3.8–29.3) higher risk of poor visual memory (45% vs 8%, *p* < 0.001). The magnitude of this atrophy was an individual predictor of poor visual memory (AUC = 0.74, *p* < 0.001), and it remained significant after controlling for HS (*p* = 0.002). This pattern was not predictive of poor verbal memory (27% vs 21%, *p* = 0.48).

In the validation cohort, only 3 patients had poor visual memory, thus not supporting enough data for model validation.

#### Memory Decline after Surgery

Eighteen of the 55 patients who underwent left ATLR had an atrophic left hippocampal tail (see Fig 3A). This pattern was associated with a 3.9‐fold (95% CI = 1.1–14.6) higher risk of postsurgical verbal memory decline below the 5th percentile on an RCI compared to those without (44% vs 19%, *p* = 0.04). This pattern was predictive at an individual level (AUC = 0.69, *p* = 0.03) but was not significant when controlling for HS (odds ratio = 2.5, 95% CI = 0.7–9.8, *p* = 0.18). There were no shape abnormalities predictive of visual memory decline.

In the validation cohort, patients with (n = 3) compared to those without (n = 21) significant atrophy of the left hippocampal tail had a 2.1‐fold (95% CI = 0.2–29.7; 33% vs 19%) higher risk of postsurgical verbal memory decline with an AUC of 0.74 (*p* = 0.04).

## Discussion

We found that distinct morphological alterations of the hippocampal surface that were common to both LTLE and RTLE correlated with verbal and visual memory performance. Atrophy of the superolateral side of the left hippocampal head was associated with poor verbal memory (see Fig 1A). Poor visual memory was associated with bilateral atrophy of the inferomedial hippocampus (see Fig 1B). Atrophy of the left hippocampal tail increased the risk of verbal memory decline after left temporal lobe resection (see Fig 3A). We validated the hippocampal shape patterns predicting pre‐ and postsurgical verbal memory in an independent cohort of unilateral TLE cases.

We demonstrated distinct patterns of hippocampal atrophy that were associated with a higher risk of memory deficits before and after epilepsy surgery, supporting a subregionally specific representation of certain memory functions in the hippocampus. Some of our findings argue against a traditional fully lateralized model of material‐specific memory and rather support a more bilateral representation of memory function (see Fig 1B, D, E).

### Verbal Memory

Poor verbal memory was associated with atrophy of the superolateral head of the left hippocampus irrespective of TLE lateralization (see Fig 1A). This most likely corresponds to atrophy in the anterior CA1 subfield. Numerous studies,^11^, ^12^, ^14^, ^16^, ^17^, ^18^, ^20^ but not all previous histopathological investigations,^9^, ^10^ have reported a correlation between neuronal cell loss in the CA1 subfield and preoperative verbal memory. Studies in nondemented elderly showed an association between verbal memory and hippocampal head size.^39^

The left hippocampal head is included in a standard left ATLR (see Fig 3A left). This is consistent with the finding of frequent verbal memory impairment after left ATLR and could explain why such deficits are less common after right temporal resections (RCI: left 27% vs right 7%; see Table 1).

Worsened verbal memory after left anterior temporal lobe removal was predicted by atrophy of the left hippocampal tail (see Fig 3A), which is not resected during standard ATLR. A memory‐fMRI study showed increased activation of the hippocampal tail in patients whose memory improves following surgery. Increased activation in the remnant hippocampal tail compared to preoperatively was seen 3 months after surgery in those with declining memory function, suggesting compensatory but inefficient neuronal plasticity.^40^, ^41^ Another study indicates that postsurgical ipsilateral recruitment of the posterior hippocampal remnant is important for preserving language.^42^ We speculate that presurgical structural disturbances of the left hippocampal tail could contribute to the above fMRI findings, leading to an impaired ability of the remnant to compensate for anterior temporal lobe removal and so impair neuronal plasticity after surgery. Oppositely, an fMRI study showed that increased preoperative verbal memory activation in the posterior left hippocampus was protective against verbal memory decline following surgery.^41^ Presurgical atrophy of the left hippocampal tail may explain failure to engage the posterior hippocampus prior to surgery on fMRI memory tasks and may represent a novel predictor of verbal memory decline after left temporal lobe removal.

### Visual Memory

Poor visual memory was associated with atrophy of the bilateral inferomedial hippocampal surface in the overall group of all TLE patients (see Fig 1B). This is in accordance with recent fMRI evidence for a more bilateral representation of visual memory.^43^ Such a bilateral representation of visual memory within both hippocampi could explain why patients after both left and right temporal lobe resections are at similar risk of visual memory decline (RCI: left 9% vs right 6%; see Table 1). We speculate that due to a bilateral visual memory representation, the unaffected contralateral hippocampus might compensate for resections of the ipsilateral temporal lobe. This could explain why visual memory decline was less severe and less frequent than verbal memory decline after temporal lobe resection (RCI: 17% in verbal vs 7% in visual memory; see Table 1).

Atrophy of the inferomedial hippocampal surface likely corresponds to cell loss in the subiculum (see Fig 1B). The subiculum has received little attention in previous literature on memory function, although some studies indicate this region's importance in supporting visual memory.^44^ Episodic memory fMRI studies suggested activation of the dentate gyrus and several subfields during learning, whereas the subiculum was predominantly activated during episodic memory recollection,^45^ which is key to performing well on most verbal/visual memory tests such as the AMIPB.

### Laterality of Findings

Some aspects of our findings are in support of a material‐specific segregation of memory function into dominant and nondominant hemispheres.^46^ RTLE patients had lower presurgical visual memory scores than LTLE patients (see Table 1), verbal immediate and delayed recall correlated with a cluster in the left hippocampal head in all TLE patients (see Fig 1A), and visual delayed recall was associated with atrophy of the right inferomedial hippocampus (see Fig 2). Moreover, following LTLE surgery verbal learning skill decreases, with no change in visual learning ability after RTLE surgery. On the other hand, several of our findings argue against a strict lateralization of memory functions. Both LTLE and RTLE had worse verbal memory performance than healthy controls, and there was no verbal learning difference between LTLE and RTLE groups (see Table 1). Additionally, In RTLE, verbal memory correlated with inward deformations of the right hippocampus (see Fig 1E). These results suggest a more important role for the right hippocampus in verbal memory, which is supported by significantly decreased verbal memory performance in RTLE patients compared to controls (see Table 2). In recent years, a paradigm shift toward a more bilateral framework of memory organization could be observed that is in accordance with these observations.^4^

Visual memory correlated with bilateral clusters affecting the inferomedial surface of both hippocampi (see Fig 1B, D). These findings support a predominantly, but not exclusively, left‐lateralized representation of verbal memory and a more bilateral representation of visual memory.

A novel finding was that the right hippocampus was larger in LTLE patients compared to controls (see Table 1). Previous fMRI studies described reorganization of memory function in the contralateral hemisphere in unilateral TLE patients.^43^ We hypothesize that functional compensation after disruption of the left hippocampus could lead to increased activation in the right hippocampus, eventually promoting hippocampal growth. A recent shape analysis study interestingly indicated contralateral inflation of the left hippocampus in RTLE, proposing a similar mechanism of functional compensation.^47^

### Methodological Considerations

This study has several strengths. Our data were obtained from a large cohort of well‐phenotyped TLE patients undergoing epilepsy surgery that had standardized neuropsychological testing and high‐resolution MRI data acquired on the same scanner. Hippocampal shape analysis is an objective noninvasive method with high inter‐rater reliability that can be applied to routine MRI. Shape analysis allowed us to detect novel findings that are not measurable using histopathology, that is, bilateral hippocampal changes in visual memory deficits (see Fig 1B) and the prognostic role of hippocampal tail abnormalities in postsurgical verbal memory decline (see Fig 3A). Comparable results were observed in immediate (see Fig 1) and delayed (see Fig 2) recall, adding further support to our findings. Hippocampal shape patterns can be determined noninvasively before surgery and can thus support presurgical neuropsychological counseling and surgical planning. We successfully validated the hippocampal shape patterns predicting pre‐ and postsurgical verbal memory in an independent cohort, supporting the generalizability of these results.

Our study has several limitations. Analyses of postsurgical outcome were restricted to data available for 109 subjects. We did not have a sufficiently large and severely affected validation cohort of patients with poor visual memory (n = 3). Thus, hippocampal shape patterns predictive of poor visual memory need to be interpreted with caution, and validation in a larger cohort is warranted. Our analysis was restricted to the hippocampal surface, and hippocampal subfields hidden in depth (CA4) could not be explored. We provided heuristic subfield boundaries on our hippocampal figures, but these approximations need to be interpreted with caution. A limitation inherent to all studies in refractory TLE patients undergoing epilepsy surgery is that patients are treated with antiepileptic drugs and the influence of medication on cognition cannot be eliminated.

Not all morphological results found in the combined epilepsy group translated to the LTLE and RTLE subgroups. Some of these differences can be attributed to a reduced power to detect significant results in a smaller group of subjects. Several effects and trends seen on T‐value maps in these subgroups implicate regions similar to those found in the overall patient group. Similar trends compared to the overall group were observed in relation to verbal and visual memory in LTLE (see Fig 1C, D) and to visual memory in RTLE (see Fig 1F). However, important differences should also be noted. Verbal memory in RTLE correlated with medial and lateral atrophy of the right hippocampus. This argues against the traditional material‐specific model of memory.^4^ It also highlights that relevant differences in memory representation and processing might exist between patients with LTLE and RTLE. Larger studies will need to explore these differences in the future.

### Conclusions

Hippocampal surface‐shape analysis can demonstrate hippocampal morphology and its impact on cognitive function, which is not captured by global volume measurements. Our results suggest a subregionally specific representation of memory functions in the hippocampus, which will be relevant in view of recently developed highly selective surgical procedures (ie, laser‐induced thermal therapy or magnetic resonance–guided focused ultrasound).^48^ TLE is not a homogeneous disease but rather a disorder with different subtypes.^36^ We extend these findings by segregating TLE patients into subgroups based on their hippocampal shape patterns showing that these patterns correlate with the risk of presurgical and postsurgical memory deficits. This has practical implications for neuropsychological counseling of people with epilepsy.

## Author Contributions

T.S.P., S.B., P.J.T., M.K.S., S.B.V., M.T., J.S.D., M.J.K., and M.G. contributed to the conception and design of the study; T.S.P., C.C.‐L., I.C., J.d.T., J.L.B., L.C., G.P.W., S.B.V., J.S.D., M.J.K., and M.G. contributed to the acquisition and analysis of data; T.S.P., C.C., I.C.‐L., M.J.K., and M.G. contributed to drafting the text and preparing the figures.

## Potential Conflicts of Interest

Nothing to report.

## Supporting information

**TABLE S1.** Review of Quantitative Histology Studies Analyzing the Association of Hippocampal Subfield Neuronal Counts and Verbal/Visual Memory
**TABLE S2.** Association of Pathology with Presurgical Memory Performance
**TABLE S3.** Association of Atypical Language Lateralization with Pre‐ and Postsurgical Memory Scores in LTLE

## Endnote

c Significant (*p* < 0.05) difference compared to contralateral TLE
