# Supplementary material for: Hippocampal Shape Is Associated with Memory Deficits in Temporal Lobe Epilepsy
Source: Ann Neurol. 2020 May 28;88(1):170–82. doi: 10.1002/ana.25762 (PMC8432153; doi:10.1002/ana.25762)
Supplement: Supplementary file 1 — TABLE S1. Review of Quantitative Histology Studies Analyzing the Association of Hippocampal Subfield Neuronal Counts and Verbal/Visual Memory TABLE S2. Association of Pathology with Presurgical Memory Performance TABLE S3. Association of Atypical Language Lateralization with Pre‐ and Postsurgical Memory Scores in LTLE [file ANA-88-170-s001.docx]

**Supplementary Table 1:** *Review of quantitative histology studies analysing the association of hippocampal subfields neuronal counts and verbal/visual memory.*

| **Study** | **Participants** | **Analysed regions/subfields** | **Outcome** | **Conclusions** |
| --- | --- | --- | --- | --- |
| Sass et al. 1990 | TLE (n=35),  L (n=18), R (n=17) | CA1, CA2, CA3, the hilar area, granule cell layer of the area dentata | vSRT | Association between neuronal density in CA3 and the hilar area in the dominant hemisphere and presurgical verbal memory impairment. |
| Sass et al. 1991 | TLE (n= 46),  L (n=21), R (n=25) | CA1, CA2, and CA3; the hilum; and the dentate granule cell layer | Brief memory assessment during IAP | Association between neuronal density in CA3 and memory scores. |
| Sass et al. 1992 | TLE (n=59),  L (n=28), R (n=31) | CA1, CA2, CA3, the hilar area and the granular layer of area dentata | BNT, WAIS-R, WMS | Association between neuronal density in CA3 and the hilar area with percent retention scores in left TLE. |
| O'Rourke et al. 1993 | TLE (n=23) | CA1, CA2, and CA3; the hilum; and the dentate granule cell layer | Brief memory assessment during IAP | Association between neuronal density in the hilar and dentate granule regions with the degree of unilateral memory impairment ipsilateral to the seizure focus. |
| Sass et al. 1995 | TLE with structural lesions (n=22) | CA1, CA2, CA3, the hilar area, and the granule layer of area dentata | vSRT, WMS | Association between neuronal density in CA1 and Longlong term retrieval. |
| Baxendale et al. 1998 | TLE-HS (n=47),  L (n=24), R (n=24) | CA1, CA4 | WAIS-R, AMIBP, GNT | Association between neuronal density in CA1 in the left hemisphere with scores on immediate and delayed recall of story. |
| Zentner et al. 1999 | TLE (n=40),  L (n=20), R (n=20) | CA1–CA4 sectors, dentate fascia, and subiculum | Verbal memory: VLMT, Visual memory: DCS-R | Association between neuronal density of left CA1, subiculum, and dentate gyrus with verbal memory performance (learning capacity, recall after delay, and recognition). CA2, CA3, and CA4 correlated with visual recognition. |
| Pauli et al. 2006 | TLE (n=24) | CA1, CA2, and CA3; the hilum; and the dentate granule cell layer | Brief memory assessment during IAP | Association between neuronal density of the dentate gyrus, CA1, and CA3 with memory scores. |
| Coras et al. 2014 | TLE (n=100),  L (n=45), R (n=55) | CA1, CA2, CA3 and CA, the internal and external limbs of the DG, subiculum | Berlin Amnesia Test | Association between neuronal loss affecting all hippocampal subfields (HS ILAE Type 1) and predominant cell loss in CA4 partially affecting also CA3 and DG (HS ILAE Type 3) with reduced declarative memory. No association between CA1 predominant cell loss (HS ILAE Type 2) with declarative memory. |
| Witt et al. 2014 | mTLE (n= 104),  L (n=53), R (n=49) | CA1, CA2, CA3, CA4, internal and external limb of the DG | Verbal memory: VLMT, Visual memory: DCS-R | The overall pathological status of the left hippocampus correlated with verbal memory scores. A superior role of a single hippocampal subregion or cell loss pattern was not found. |
| Comper et al. 2017 | mTLE (n =72),  L (n=36), R ( n=36) | CA1, CA2, CA3, CA4 | RAVLT and Logical Memory, Complex Rey Figure | Association between neuronal density of left CA1 with Logical Memory and RAVLT delayed recall. Association between right CA1 subfield and Complex Rey Figure immediate recall scores. |

TLE= temporal lobe epilepsy, L = left, R= right, HS=Hippocampal sclerosis, CA= cornu ammoni, DG = Dentate Gyrus, ERC= entorhinal cortex, vSRT= verbal selective reminding test, WAIS-R= Wechsler Adult Intelligence Scale-Revised, AMIBP= Adult Memory and Information Processing Battery, DCS-R= Diagnosticum für Cerebralschädigung, VLMT=Verbaler Lern- und Merkfähigkeitstes, which represents a German equivalent to the Rey auditory verbal learning test, RAVLT= Rey Auditory Verbal Learning Test, BNT= Boston naming test, WMS= Wechsler Memory Scale, GNT= graded naming test, RBANS= Repeatable Battery for the Assessment of Neuropsychological Status, DGint= internal limb of the dentate gyrus, DGext= external limb of the dentate gyrus, ILAE= International League Against Epilepsy.

**Supplementary Table 2:** *Association of pathology with presurgical memory performance.*

|  | **All TLE** | | | **LTLE** | | | **RTLE** | | |
| --- | --- | --- | --- | --- | --- | --- | --- | --- | --- |
|  | **Non-lesional**  **(n=112)** | **Lesional**  **(n=33)** | **P value** | **Non-lesional**  **(n=58)** | **Lesional**  **(n=18)** | **P value** | **Non-lesional**  **(n=54)** | **Lesional**  **(n=15)** | **P value** |
| Verbal memory | 47.2 ± 11.8 | 47.0 ± 12.5 | 0.94 | 45.7 ± 12.1 | 44.4 ± 13.3 | 0.70 | 48.8 ± 11.4 | 50.2 ± 11.1 | 0.68 |
| Visual memory | 33.1 ± 8.4 | 35.2 ± 6.8 | 0.20 | 34.2 ± 8.2 | 37.2 ± 5.3 | 0.15 | 32.0 ± 8.6 | 32.8 ± 7.8 | 0.74 |
| Verbal memory recall | 9.3 ± 3.8 | 9.3 ± 3.6 | 0.95 | 8.5 ± 4.3 | 8.2 ± 3.9 | 0.84 | 10.2 ± 3.1 | 10.7 ± 2.8 | 0.59 |
| Visual memory recall | 6.5 ± 2.8 | 7.1 ± 2.1 | 0.27 | 6.6 ± 2.8 | 7.4 ± 1.6 | 0.26 | 6.4 ± 2.8 | 6.7 ± 2.6 | 0.70 |

Non-lesional cases include cases with hippocampal sclerosis or normal pathology/MRI. Lesional cases include patients with lesions other than hippocampal sclerosis.

Data on language lateralization was determined using language fMRI and was available in 59/76 (78%) of LTLE cases. In 48/59 (81%) LTLE patients the left hemisphere was language dominant, in 11/59 (11%) language was atypical lateralized. There were no differences in presurgical memory performance between groups with typical and atypical language lateralization. Although patients with atypically localized language had a slightly smaller risk of verbal memory decline after surgery (45% vs. 59%), the difference was not significant (p=0.27). See Supplementary Table 2 for details.

**Supplementary Table 3:** *Association of atypical language lateralization with pre- and postsurgical memory scores in LTLE.*

|  | **LTLE with left dominant language**  **(n=48)** | **LTLE with atypical language lateralisation**  **(n=11)** | **P value** |
| --- | --- | --- | --- |
| **Neuropsychometry before surgery** | |  |  |
| Verbal memory | 45.0 ± 10.0 | 47.4 ±11.1 | 0.30 |
| Visual memory | 31.4 ± 8.0 | 33.2 ± 6.9 | 0.29 |
| Verbal memory recall | 8.6 ± 3.6 | 9.0 ± 3.1 | 0.62 |
| Visual memory recall | 6.4 ± 2.6 | 6.6 ± 3.0 | 0.81 |
| **Neuropsychometry 1 year after surgery (data in 86 subjects)** | | | |
| Change in verbal memory | -4.5 ± 10.5 | -1.7 ± 13.0 | 0.32 |
| Change in visual memory | -1.6 ± 6.9 | -2.7 ± 6.4 | 0.54 |
| RCI decline verbal memory | 48 (59%) | (45%) | 0.27 |
| RCI decline visual memory | 11 (17%) | 3 (15%) | 0.86 |
